# Attributes of errors, facilitators, and barriers related to rate control of IV medications: a scoping review

**DOI:** 10.1186/s13643-023-02386-z

**Published:** 2023-12-13

**Authors:** Jeongok Park, Sang Bin You, Gi Wook Ryu, Youngkyung Kim

**Affiliations:** 1 College of Nursing, Mo-Im Kim Nursing Research Institute, Yonsei University, Seoul, Korea; 2 University of Pennsylvania School of Nursing, Philadelphia, PA USA; 3 Department of Nursing, Hansei University, 30, Hanse-Ro, Gunpo-Si, 15852 Gyeonggi-Do Korea; 4 College of Nursing and Brain Korea 21 FOUR Project, Yonsei University, Seoul, Korea

**Keywords:** Medication safety, Nurses, Patient safety, Quality improvement, Safety culture

## Abstract

**Background:**

Intravenous (IV) medication is commonly administered and closely associated with patient safety. Although nurses dedicate considerable time and effort to rate the control of IV medications, many medication errors have been linked to the wrong rate of IV medication. Further, there is a lack of comprehensive studies examining the literature on rate control of IV medications. This study aimed to identify the attributes of errors, facilitators, and barriers related to rate control of IV medications by summarizing and synthesizing the existing literature.

**Methods:**

This scoping review was conducted using the framework proposed by Arksey and O’Malley and PRISMA-ScR. Overall, four databases—PubMed, Web of Science, EMBASE, and CINAHL—were employed to search for studies published in English before January 2023. We also manually searched reference lists, related journals, and Google Scholar.

**Results:**

A total of 1211 studies were retrieved from the database searches and 23 studies were identified from manual searches, after which 22 studies were selected for the analysis. Among the nine project or experiment studies, two interventions were effective in decreasing errors related to rate control of IV medications. One of them was prospective, continuous incident reporting followed by prevention strategies, and the other encompassed six interventions to mitigate interruptions in medication verification and administration. Facilitators and barriers related to rate control of IV medications were classified as human, design, and system-related contributing factors. The sub-categories of human factors were classified as knowledge deficit, performance deficit, and incorrect dosage or infusion rate. The sub-category of design factor was device. The system-related contributing factors were classified as frequent interruptions and distractions, training, assignment or placement of healthcare providers (HCPs) or inexperienced personnel, policies and procedures, and communication systems between HCPs.

**Conclusions:**

Further research is needed to develop effective interventions to improve IV rate control. Considering the rapid growth of technology in medical settings, interventions and policy changes regarding education and the work environment are necessary. Additionally, each key group such as HCPs, healthcare administrators, and engineers specializing in IV medication infusion devices should perform its role and cooperate for appropriate IV rate control within a structured system.

**Supplementary Information:**

The online version contains supplementary material available at 10.1186/s13643-023-02386-z.

## Background

Medication errors are closely associated with patient safety and the quality of care [1, 2]. In particular, medication errors, which denote a clinical issue of global importance for patient safety, negatively affect patient morbidity and mortality and lead to delays in discharge [3, 4]. The National Health Service in the UK estimates that 237 million medication errors occur each year, of which 66 million cause clinically significant harm [5]. The US Food and Drug Administration reported that they received more than 100,000 reports each year associated with suspected medication errors [6]. Additionally, it was estimated that 40,000–98,000 deaths per year in the USA could be attributed to errors by healthcare providers (HCPs) [7]. Previous studies have revealed that medication errors account for 6–12% of hospital admissions [8].

Intravenous (IV) medication is a common treatment in hospitalized patient care [9]. It is used in wards, intensive care units (ICUs), emergency rooms, and outpatient clinics in hospitals [9, 10]. As direct HCPs, nurses are integral in patient safety during the IV medication process which could result in unintended errors or violations of recommendations [3]. As many drugs injected via the IV route include high-risk drugs, such as chemotherapy agents, insulin, and opioids [10], inappropriate dose administration could lead to adverse events (AEs), such as death and life-threatening events [11, 12].

IV medication process is a complex and multistage process. There are 12 stages in the IV medication process, which can be classified as follows: (1) obtain the drug for administration, (2) obtain the diluent, (3) reconstitute the drug in the diluent, (4) take the drug at the patient’s bedside, (5) check for the patient’s allergies, (6) check the route of drug administration, (7) check the drug dose, (8) check the patency of the cannula, (9) expel the air from the syringe, (10) administer the drug, (11) flush the cannula, and (12) sign the prescription chart [13]. IV medication errors can occur at any of these stages. It is imperative to administer the drug at the correct time and rate during the IV medication process [13]. The National Coordinating Council for Medication Error Reporting and Prevention (NCC MERP) defined an error in IV medication rates as “too fast or too slow rate than that intended” [14]. Maintaining the correct rate of IV medication is essential for enhancing the effectiveness of IV therapy and reducing AEs [9].

Infusion pumps are devices designed to improve the accuracy of IV infusions, with drug flow, volume, and timing programmed by HCPs [15]. A smart pump is an infusion pump with a software package containing a drug library. During programming, the smart pump software warns users about entering drug parameters that deviate from the recommended parameters, such as the type, dose, and dosage unit of the drug [15]. In the absence of a device for administering IV medication, such as an infusion pump or smart pump, the IV rate is usually controlled by counting the number of fluid drops falling into the drip chamber [9].

According to the previous study, applying an incorrect rate was the most prevalent IV medication error, accounting for 536 of 925 (57.9%) total IV medication errors [16]. Although rate control of IV medications is critical to patient safety and quality care, few studies review and map the relevant literature on rate control of IV medications. Therefore, this study aimed to identify the attributes of errors, facilitators, and barriers related to rate control of IV medications by summarizing the existing literature.

The specific research questions of this study are as follows:

1. What are the general characteristics of the studies related to rate control of IV medications?
2. What are the attributes of errors associated with rate control of IV medications?
3. What are the facilitators and barriers to rate control of IV medications?

## Methods

This scoping review followed the framework suggested by Arksey and O’Malley [17] and developed by Levac et al. [18] and Peters et al. [19]. Preferred Reporting Items for Systematic Reviews and Meta-Analyses extension for Scoping Reviews (PRISMA-ScR) developed in 2020 by the Joanna Briggs Institute (JBI) were used to ensure reliability in the reporting of methodology (Additional file 1) [19].

### Search strategy

According to the JBI Manuals for Evidence Synthesis, a three-step search strategy was adopted [19]. First, a preliminary search in PubMed was conducted based on the title, abstract, keywords, and index terms of articles to develop our search strategy. In the preliminary search, we used keywords such as “patients,” “nurse,” “IV therapy,” “monitoring,” “rate,” and “medication error.” The search results indicated that studies on medical devices and system-related factors were excluded. Therefore, we decided to exclude the keywords “patients” and “nurse” and focus on “IV therapy,” “monitoring,” “rate,” and “medication error” to comprehensively include studies on factors associated with rate control of infusion medications. Secondly, we used all identified keywords and index terms across all included databases following consultations with a research librarian at Yonsei University Medical Library to elaborate our search strategy. Four databases—PubMed, CINAHL, EMBASE, and Web of Science—were searched using the keywords, index terms, and a comprehensive list of keyword variations to identify relevant studies published before January 2023. The details of the search strategy are described in Additional file 2. All database search results were exported into Endnote version 20. Finally, we manually searched the reference lists of the included articles identified from the database search. Furthermore, we manually searched two journals related to medication errors and patient safety, and Google Scholar to comprehensively identify the relevant literature. When performing a search on Google Scholar, keywords such as “medication,” “rate,” “IV therapy,” “intravenous administration,” and “medication error” were appropriately combined using search modifiers.

### Eligibility criteria

Inclusion criteria were established according to the participants, concept, and context (PCC) framework recommended by the JBI manuals for scoping reviews [19]. The participants include patients receiving IV therapy, HCPs involved in administering IV medications, and experts from non-healthcare fields related to rate control of IV medications. The concepts were facilitators and barriers to rate control of IV medications, and the contexts were the environments or situations in which errors in rate control of IV medications occurred. While screening the literature identified by the three-step search based on the inclusion criteria, we refined the exclusion criteria through discussion among researchers. The exclusion criteria were as follows: (1) not available in English, (2) not an original article, (3) studies of medication errors in general, (4) not accessible, or (5) prescription error.

### Study selection

Once duplicates were automatically removed through Endnote, two independent researchers assessed the eligibility of all articles by screening the titles and abstracts based on the inclusion and exclusion criteria. Studies identified via database searches were screened by GWR and YK and studies identified via other methods were screened by SBY and YK. Full-text articles were obtained either when the studies met the inclusion criteria or when more information was needed to assess eligibility and the researchers independently reviewed the full-text articles. In case of any disagreement in the study selection process, a consensus was reached through discussion among three researchers (GWR, SBY, and YK) and a senior researcher (JP).

### Data extraction

Through consensus among the researchers, a form for data extraction was developed to extract appropriate information following the JBI manuals for scoping reviews [19]. The following data were collected from each study: author information, publication year, country, study design, study period, aims, participants or events (defined as the occurrences related to patient care focused on in the study), contexts, methods, errors related to the control of IV medications (observed results or intervention outcomes), error severity, facilitators, and barriers according to the NCC MERP criteria. Three researchers (GWR SBY, and YK) independently conducted data charting and completed the data extraction form through discussion.

### Data synthesis

The general characteristics of included studies such as publication year, country, study design, and study period were analyzed using descriptive statistics to identify trends or patterns. The aims, participants, events, contexts, and methods of the included studies were classified into several categories through a research meeting including a senior researcher (JP) to summarize and analyze the characteristics of the included studies comprehensively. Attributes of errors associated with rate control of IV medications were analyzed and organized through consensus among researchers based on extracted data. Facilitators and barriers to rate control of IV medications were independently classified according to NCC MERP criteria by three researchers (GWR, SBY, and YK) and iteratively modified. Discrepancies were resolved by discussion and re-reading the articles, with the final decision made in consultation with the senior researcher (JP).

## Results

### Study selection

**Fig. 1:**
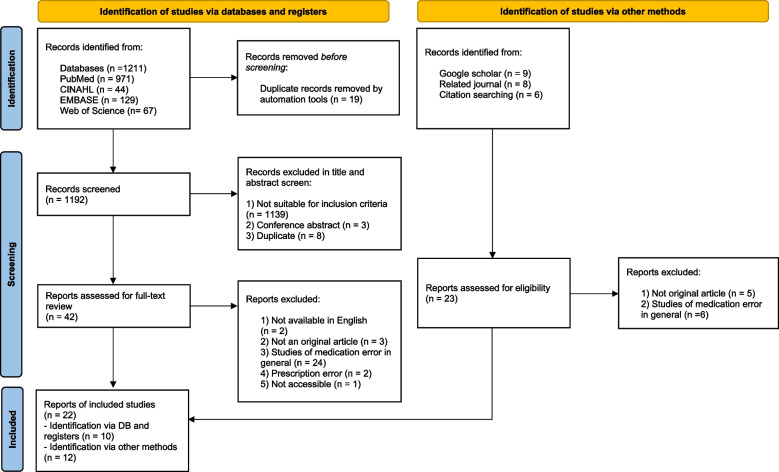
PRISMA flow chart for literature selection

A total of 1211 studies were selected through a database search. After reviewing the titles and abstracts of the studies, 42 studies were considered for a detailed assessment by the three researchers. In particular, 2 were not available in English, 3 were not original articles, 24 were studies of medication error in general without details on rate control of IV medications, 2 were regarding prescription errors, and 1 was not accessible. Finally, 10 studies were identified through a database search. Additionally, 23 studies were identified from a manual search. Among the 23, 5 were not original articles, and 6 were studies on medication error in general. Finally, 12 studies were identified via other methods. Hence, 22 studies were included in the data analysis (Fig. 1, Additional file 3).

### Characteristics of the studies

#### General characteristics

Table 1 presents the general characteristics of the included studies. Two of the included studies had a publication year before 2000 [20, 21], and more than half of the studies (*n* = 15) were published in 2010 and later. A majority of the included studies were conducted in Western countries (*n* = 15) [22–36], four were conducted in Asia [20, 37–39], two were conducted in Australia [21, 40], and one was conducted in Egypt [2]. In terms of the study design, most studies were project studies (*n* = 7) [22, 24, 27, 28, 30, 34, 39] or prospective observational studies (*n* = 5) [2, 20, 29, 32, 40], followed by retrospective studies (*n* = 3) [21, 25, 35], qualitative or mixed-methods studies (*n* = 3) [23, 26, 33], and descriptive cross-sectional studies (*n* = 2) [36, 38]. Additionally, there was one controlled pre-posttest study [37] and one simulation laboratory experiment study [31]. The study period also varied greatly from 2 days [32] to 6 years [25].

**Table 1:** Characteristics of the included studies (*n* = 22)

First author (year)/country	Study design/study period	Aims	Participants/events	Context	Methods (data collection or intervention)
Short (1993) [20]/Hong Kong	Prospective observational study/1 year	To find out the cause related to anesthetic critical events	P: anesthesiologists who reported the critical events E: 125 critical events that were reported by anesthesiologists in 16,379 anesthesia cases	A hospital (1430 beds)	D: a review of the voluntary reporting of critical events
Singleton (1993) [21]/Australia	Retrospective study/NA	To analyze the problems associated with vascular access	P: individuals who voluntarily and anonymously reported any unintended incidents that either reduced or had the potential to reduce the safety margin for a patient E: 65 events involving problems with access to the vascular system of the first 2000 incidents reported to the AIMS	Hospitals and other healthcare settings	D: a review of reporting to AIMS
Goldspiel (2000) [22]/USA	Project study/54 months	To reduce the number of chemotherapy-related medication errors	P: members from the pharmacy department, nursing department, National Cancer Institute intramural program, information system department, and hospital administration E: chemotherapy-related medication errors occurred during the study period	A hospital (325 beds)	I: PDCA (plan-do-check-act) performance improvement model with a comprehensive, interdisciplinary approach D: prescribing errors that were detected before medication preparation and medication errors that were reported through an occurrence-reporting system
Taxis (2003) [23]/UK	Qualitative study/76 days	To investigate the causes of IV medication errors in drug preparation and administration	P: 113 nurses and 1 doctor E: 265 IV medication errors in 483 drug preparation and 447 drug administration	10 wards in two hospitals	D: observation by trained pharmacists
Wetterneck (2006) [24]/USA	Project study/6 months	To describe the use of FMEA to guide the implementation of a smart IV pump	P: a multidisciplinary team which was composed of representatives from anesthesiology, biomedical engineering central supply, industrial engineering, internal medicine, nursing, pharmacy, and quality improvement E: Failure modes expected before implementation of smart IV pump and failure modes that occurred 3 months after implementation	A hospital	I: smart IV pump implementation equipped with FMEA by a multidisciplinary committee
Rinke (2007) [25]/USA	Retrospective study/6 years	To identify patterns in pediatric chemotherapy errors	P: healthcare professionals who voluntarily reported pediatric chemotherapy errors in the national medication safety reporting system E: 310 pediatric chemotherapy error reports of 829,492 errors reported to internet-accessible, anonymous adverse drug reactions and medication errors reporting program	69 healthcare facilities	D: a review of the incidents reported to the reporting program database from 1999 to 2004
Nuckols (2008) [26]/USA	Mixed methods/20,559 bed-days	To compare preventable IV ADE incidence rates between smart pumps and infusion pumps	P: 4 ~ 5 critical care nurses per hospital and 4 board-certified internal medicine physicians E: 100 preventable IV ADE among 4604 patients	ICUs in two hospitals	D: retrospective medical chart review and qualitative descriptions of errors
Evans (2010) [27]/USA	Project study/22.5 months	To evaluate the smart system that could detect pump programming errors	P: nurses and critical care clinical pharmacists E: 970 alerts on 25,040 infusion pump cases	ICU (24 beds) in one hospital (456 beds)	I: a smart system that was connected to the EMR to prevent pump programming errors D: follow-up on every pump alert by a critical care clinical pharmacist
Ligi (2010) [28]/France	Project study/4 years	To evaluate the effect of continuous incident reporting and subsequent prevention strategies on the incidence of severe iatrogenic events and targeted priorities in admitted neonates	P: staff members (physicians, senior nurses, and nurses) E: 622 iatrogenic events among 1033 neonates	Neonatal center (54 beds)	I: prospective, continuous incident reporting followed by the implementation of prevention strategies such as double-checks infusion pump programming by a multidisciplinary care quality improvement team D: voluntary incident reports from the anonymous, non-punitive system
Westbrook (2011) [40]/Australia	Prospective observational study/11 months	To explore the IV administration errors and the relationships between errors, procedural failures, and nurse experience	P: 107 nurses E: 568 IV medication administrations	6 wards across two hospitals (400 beds, 326 beds, respectively)	D: observation by 3 researchers (registered nurses or doctors)
Kandil (2012) [2]/Egypt	Prospective observational study/9 months	To find out the patterns of medication errors in an obstetric emergency ward	P: 10,000 patients E: 1976 administration errors of 47,192 medical prescriptions	An obstetric emergency ward	D: observation by head nurses and retrospective review of the patients’ charts and nurses’ notes by the authors
Rodriguez-Gonzalez (2012) [29]/Spain	Prospective observational study/1 week	To identify the frequency of medication preparation and administration errors in medical units using automated prescription and dispensing systems	P: 23 nurses and 73 patients E: 509 errors in 2314 medication administrations	Two gastroenterology units (30 and 29 beds) in one hospital (1537 beds)	D: observation by trained pharmacists and nurses
Ohashi (2013) [30]/USA	Project study/3 days	To develop a web-based IV medication error observational tool and validate the tool	P: 55 patients E: 171 errors of 181 IV medication administrations observed	Medical ICU, surgical ICU, and general surgical units in one hospital (793 beds)	I: a web-based data collection tool that was developed by an interdisciplinary team D: observation by two nurses using the Redcap
Nguyen (2014) [37]/Vietnam	Controlled pre-posttest study/5 weeks	To evaluate the effect of the training program on clinically relevant errors during IV medication preparation and administration	P: nurses in the two ICUs E: 1204 IV medication administration (516 during the baseline period and 688 during the follow-up period)	ICU (the intervention ward) and PSU (the control ward) in one hospital	I: clinical pharmacist-led training program that was developed by a clinical pharmacist and the chief nurse (classroom lectures, practice-based education, two posters and written guidelines for safe preparation and administration) D: observation by nurses
Prakash (2014) [31]/Canada	Simulation laboratory experiment study/6 months	To assess the effects of interruption on medication verification and administration errors, and the effectiveness of targeted interventions	P: 37 nurses (experimental group: 19, control group: 18) E: errors in 385 medication verification and administration tasks (pre-intervention: 252, post-intervention: 133)	Simulated ambulatory chemotherapy setting	I: verification booth, standardized workflow, speaking aloud for medication verification tasks, visual timers for IV pushes, no interruption zones with motion-activated indicators, and reminder signage for medication administration tasks D: observation by two trained observers in an observation room located behind a one-way glass
Bagheri-Nesami (2015) [38]/Iran	Descriptive cross-sectional study/2 months	To identify the frequency of IV infusion errors and their causes in cardiac critical care units	P: 190 nurses E: 262 IV medication errors	Cardiac critical care units from 12 teaching hospitals	D: self-reporting questionnaire
Schnock (2017) [32]/USA	Prospective observational study/2 ~ 4 days	To investigate the frequency and types of IV medication errors related to smart pumps	P: 478 patients E: 1691 errors of 1164 IV medication administrations	10 hospitals (one medical ICU, one surgical ICU, one general surgical unit, and one medical unit at each institution)	D: observation by two trained observers (nurse and/or pharmacist)
Tsang (2017) [39]/Hong Kong	Project study/38 months	To describe the application of the point and calling method that is a rule-based behavior composed of various aspects and evaluate the intervention to reduce the high-alert medication using infusion and syringe devices	P: about 1100 nurses who had been taught how to implement the point and calling method. One hundred forty-five DOM, WM, APN, and RN were auditors and 709 nurses were audited by them E: 5 high-alert medication incidents using infusion and syringe devices	21 wards from one hospital (1400 beds)	I: the point and calling methods on checking HAM using infusion and syringe devices developed by the workgroup comprised APNs and WMs D: questionnaires on the perception of the point and calling methods from the participating wards and incidents reporting
Lyons (2018) [33]/UK	Mixed methods/21 months	To describe the incidence, types, and severity of IV medication infusion errors	P: 1326 patients and 32 observers E: 240 infusion errors and 1491 discrepancies of 2008 prescribed IV infusion	16 NHS hospital trusts	D: observation by two trained observers (usually a nurse and pharmacist)
Schnock (2018) [34]/USA	Project study/3 years	To evaluate the preliminary effects of the infusion safety intervention bundle that was developed to decrease IV medication administration errors	P: 840 patients and 9 hospitals - Pre-intervention: 418 patients - Post-intervention: 422 patients E: 2031 IV medication administrations - Pre-intervention: 972 - Post-intervention: 1059	9 hospitals (adult medical/surgical units and medical/surgical ICUs at each institution)	I: infusion safety intervention bundle developed by a multidisciplinary research team - Labeling/IV tubing intervention, unauthorized medication intervention, drug library use intervention D: observation by two trained observers (nurse and/or pharmacist)
Taylor (2019) [35]/USA	Retrospective study/1 year	To identify the frequency of medication errors with infusion pumps and analyze the contributing factors	P: healthcare professionals who reported patient safety-related incidents and serious events in a secure, web-based system E: 1004 medication errors with infusion pumps	132 hospitals	D: a review of the events reported to the PA-PSRS during 2018
Schilling (2022) [36]/Germany	Descriptive cross-sectional study (two-phase)/6 months	▪ The first phase: to develop a German pediatric HAM list ▪ The second phase: to identify DRP and related interventions for the HAM selected in the first phase	P: 42 pharmacists - Response rate: the first phase (60%), the second phase (40%) E: 216 DRPs	NA	D: a two-step survey by mailing to the participants

*ADE* Adverse drug events, *AIMS* Australian Incident Monitoring Study, *APN* Advanced practice nurse, *D* data collection, *DOM* Department operation manager, *DRP* Drug-related problems, *E* Events, *EMR* Electronic medical records, *FMEA* Failure mode and effects analysis, *HAM* High-alert medications, *I* Intervention, *ICU* Intensive care unit *IV* Intravenous, *NA* Not applicable, *NHS* National Health Service, *P* Participants, *PA-PSRS* Pennsylvania Patient Safety Reporting System, *PSU* Post-surgical unit, *RN* Registered nurse, *WM* Ward manager

The aims of the included studies were divided into two main categories. First, 13 studies identified the current status, causes, and factors influencing errors that could occur in healthcare settings [2, 20, 21, 23, 25, 26, 29, 32, 33, 35, 36, 38, 40]. Among these, three studies were on errors that may occur in specific healthcare procedures, such as anesthesia [20], vascular access [21], and pediatric chemotherapy [25]. Additionally, three studies explored possible errors associated with specific settings and medications, such as an obstetric emergency ward [2], cardiac critical care units [38], and high-alert medications [36], and three studies investigated the errors associated with the overall IV medication preparation or administration [23, 33, 40]. Moreover, three studies aimed at identifying potential problems associated with the use of IV medication infusion devices [26, 32, 35], and one study was about errors in medication preparation and administration that could occur in a setting using a specific system connected to electronic medical records [29]. Second, nine studies described the procedure of developing interventions or identified the effect of interventions [22, 24, 27, 28, 30, 31, 34, 37, 39].

#### Participants and events

Participants in the 22 studies included HCPs such as nurses, doctors, pharmacists, and patients. Notably, four of these studies were only for nurses [31, 37, 38, 40] and there was also one study involving only pharmacists [36]. Furthermore, there were five studies wherein people from various departments or roles participated [23, 26–28, 39]. There were three studies wherein the patients were participants, and two studies included both patients and medical staff [29, 33].

Among the included studies, nine studies focused on errors in IV medication preparation and administration as events [23, 26, 30, 32–34, 37, 38, 40] and five studies focused on the administration process only [30, 32, 34, 37, 40]. Four studies focused on problems in the administration of all types of drugs including errors associated with rate control of IV medications [2, 22, 28, 29]. Additionally, four studies focused on events that occurred with IV medication infusion devices [24, 27, 35, 39], two studies explored the events that occurred during chemotherapy [22, 25], and some analyzed events with problems in vascular access [21], iatrogenic events among neonates [28], and critical events in anesthesia cases [20].

#### Contexts and methods

The contexts can be largely divided into healthcare settings, including hospitals and laboratory settings. Three hospital-based studies were conducted in the entire hospital [20, 22, 24], eight studies were conducted at several hospitals, and the number of hospitals involved varied from 2 to 132 [23, 26, 32–35, 38, 40]. Furthermore, four studies were conducted in different departments within one hospital [29, 30, 37, 39], three studies were conducted in only one department [2, 27, 28], two studies considered other healthcare settings and were not limited to hospitals [21, 25], and one study was conducted in a simulation laboratory setting that enabled a realistic simulation of an ambulatory chemotherapy unit [31].

Specifically, seven out of the nine studies developed or implemented interventions based on interdisciplinary or multidisciplinary collaboration [22, 24, 28, 30, 34, 37, 39]. Two studies developed and identified the effectiveness of interventions that created an environment for nurses to improve performance and correct errors associated with medication administration [31, 39], and two intervention studies were on error reporting methods or observation tools and the processes of addressing reported errors [28, 30]. There were also a study on a pharmacist-led educational program for nurses [37], a comprehensive intervention from drug prescription to administration to reduce chemotherapy-related medication errors [22], infusion safety intervention bundles [34], the implementation of a smart IV pump equipped with failure mode and effects analysis (FMEA) [24], and a smart system to prevent pump programming errors [27].

Data collection methods were classified as a review of reported incidents [20–22, 25, 35], a review of medical charts [26], observations [23, 29–34, 37, 40], follow-up on every pump alert [27], and self-reporting questionnaires or surveys [36, 38]. Some studies utilized retrospective reviews of reported incidents and self-report questionnaires [39]. Also, in the study by Kandil et al., observation, nursing records review, and medical charts review were all used [2].

### Attributes of errors associated with rate control of IV medications

Table 2 presents the attributes of errors related to rate control of IV medications in observed results or intervention outcomes, and error severity. Notably, 6 of 13 studies presenting observed results reported errors related to IV medication infusion devices among the rate control errors [20, 25, 32, 33, 35, 36]. Additionally, four studies reported errors in bolus dose administration or IV push and flushing lines among IV rate errors [2, 23, 36, 40]. Among the 13, nine studies reported error severity, and among these, three studies used NCC MERP ratings [25, 32, 33]. In four studies, error severity was reported by describing several cases in detail [2, 21, 23, 25], and two studies reported no injuries or damages due to errors [26, 29]. Among the nine studies that developed interventions and identified their effectiveness, four presented the frequency of incorrect rate errors as an outcome variable [28, 30, 34, 37]. Moreover, two studies suggested compliance rates for intervention as outcome variables [24, 31].

**Table 2:** Errors related to rate control of intravenous (IV) medications (*n* = 22)

First author (year)	Observed results or intervention outcomes	Error severity
Short (1993) [20]	▪ Anesthetic critical events due to syringe pump failure (*n* = 2, 1.6%)	NA
Singleton (1993) [21]	▪ Incidents with vascular system access related to the administration of drugs at an unintended rate (*n* = 2, 3%)	- Prompt collapse of the veins - Rapid onset of anesthesia - Retrograde flow of blood from the patient
Goldspiel (2000) [22]	▪ Among the 23 modifications suggested by the task force, 2 modifications were related to the infusion pump; standardize portable pumps used throughout hospital, develop policy and procedure for standardizing overfill for infusion pump preparations	NA
Taxis (2003) [23]	▪ Giving bolus doses too quickly (*n* = 168, 63.4%)	- Additional midazolam and bolus dose of adrenaline administration due to delay of continuous adrenaline infusion
Wetterneck (2006) [24]	▪ There were 18 problems after implementing the smart IV pump ▪ Two weeks after implementation, a 475-infusion audit found the drug library was used in 99.6% of medication infusions, channel labels in 80%, and the correct profile in 97% ▪ Six weeks later, in a 485-infusion audit, 99.6% of medication infusions used the drug library, 76% used channel labels, and 96% had the correct profile ▪ Approximately, 3 dosing alerts per day resulted in reprogrammed doses, which prevented potential pump programming errors from reaching patients	Temporary harm or no harm
Rinke (2007) [25]	▪ Errors in equipment and medication delivery devices (*n* = 68 of 547 possible error causes, 12.4%) ▪ Two medications were hung at the same time for the same patient, and their infusion rates were reversed (*n* = 1 of 310 error reports, 0.3%)	NCC MERP severity ratings: E (the case of reversed infusion rates)
Nuckols (2008) [26]	▪ Wrong rate (*n* = 1, 1.0%) ▪ Wrong duration (*n* = 4, 4.0%)	No injuries were observed
Evans (2010) [27]	▪ Of the 970 alerts, 137 prevented potential harm to the patient (14%)	NA
Ligi (2010) [28]	▪ Tenfold infusion rate errors per 100 admissions decreased from 2.3 to 0.6 (*p* = 0.022)	NA
Westbrook (2011) [40]	▪ Wrong rate (*n* = 266, 73.3% of four types of error; wrong mixture, wrong volume, wrong rate, or drug incompatibility) - Number of errors rated as serious of IV error type “wrong rate” (*n* = 95, 35.7%) ▪ IV administrations performed via bolus had higher error rates than infusions (*p* < 0.0001) and also higher serious error rates (*p* < 0.0001) ▪ All 72 serious errors with bolus IV infusions involved an incorrect rate ▪ 23 of 28 serious errors with IV infusions involved an incorrect rate	Serious errors mean errors that classified 3 (medium risk) and 4 (low risk) in potential Severity Assessment Code by New South Wales Health Department
Kandil (2012) [2]	▪ Wrong rate (*n* = 756, 38.3%) - Oxytocin: 926 (wrong rate, time and dose) - Misoprostol + oxytocin: 49 (wrong rate) - IV tocolytics: 41 (wrong rate) - Magnesium sulfate: 24 (wrong rate) ▪ Rates of infusion were usually set up faster than the rate prescribed or the bolus dose of medications was not administered as slowly as recommended	- Uterine hyper-contractility (oxytocin) - Monitoring for toxicity (tocolytics) - Cesarean section due to persistent fetal distress in 3 cases (oxytocin + misoprostol)
Rodriguez-Gonzalez (2012) [29]	▪ Wrong infusion speed (*n* = 27, 1.2% of all medication administration)	- No damage, but monitoring required
Ohashi (2013) [30]	▪ Clamp closed (*n* = 2, 1.1%) ▪ Incorrect rate setting in pump (*n* = 1, 0.6%) ▪ Rate deviations (*n* = 1, 0.6%) ▪ Redcap, a web-based data collection tool, was easy to use and capture IV medication errors	NCC MERP severity ratings: C (due to the errors with rate deviation)
Nguyen (2014) [37]	▪ Administration errors were approximately similar in both pre-intervention and intervention periods (based on 95% CI) - Baseline: 44.9% (CI: 38.6, 51.2) - Follow-up: 46.9% (CI: 42.1, 51.7) ▪ The prevalence of wrong administration techniques which included rate errors in the intervention ward - Baseline: 44.9% (CI: 38.6, 51.2) - Follow-up: 46.9% (CI: 42.1, 51.7) ▪ The prevalence of wrong administration techniques which included rate errors in the control ward - Baseline: 61.4% (CI: 55.7, 67.1) - Follow-up: 46.9% (CI: 48.3, 59.9)	NA
Prakash (2014) [31]	▪ Intervention utilization - For interventions that required active use by participants, the rate of utilization is as follows: - Visual timers for IV pushes: 100% - Speaking aloud during pump programming: 53% - Speaking aloud during patient identification verification: 74% ▪ Errors in IV push were significantly decreased (*p* = 0.001) ▪ Errors in pump programming and infusion initiation were significantly decreased (*p* = 0.017)	NA
Bagheri-Nesami (2015) [38]	▪ Wrong infusion rate (*n* = 45, 17.2%)	NA
Schnock (2017) [32]	▪ Smart pump use errors (*n* = 120, 10.3%): bypassing the smart pump (*n* = 16), bypassing the available drug library (*n* = 104) ▪ Wrong rate (*n* = 54, 4.6%): medications of fluids were infused slower or faster than the ordered rate (*n* = 47), the rate was set outside the titration parameter range specified in the order (*n* = 7)	NCC MERP severity rating: - Smart pump use errors: A (*n* = 4), B (*n* = 4), C (*n* = 112) - Wrong rate: A (*n* = 3), C (*n* = 49), D (*n* = 2)
Tsang (2017) [39]	▪ HAM incident related to device setting - Before the point and calling implementation (24 months): 20% - During the point and calling implementation (6 months): 17% - After the point and calling implementation (8 months): 13%	NA
Lyons (2018) [33]	▪ Rate deviation (*n* = 152, 7.6% of all infusions) ▪ Drug library not used or incorrectly used (in the case of smart pumps) (*n* = 67, 3.3% of all infusions) ▪ No significant difference in error rates between doses given via a drug library and those given without ▪ Infusions delivered with smart pumps had higher discrepancy rates than infusions delivered with conventional pumps (*p* < 0.001)	NCC MERP severity rating: ▪ Rate deviation: A (*n* = 75), C (*n* = 65), D (*n* = 12) ▪ Drug library not used or incorrectly used (in the case of smart pumps): A (*n* = 67)
Schnock (2018) [34]	▪ Smart pump/drug library not used - Pre-intervention (*n* = 114, 11.7%), post-intervention (*n* = 121, 11.4%) ▪ Wrong rate - Pre-intervention (*n* = 50, 5.1%), post-intervention (*n* = 23, 2.2%) ▪ Pump setting error - Pre-intervention (*n* = 5, 0.5%), post-intervention (*n* = 6, 0.6%) ▪ Most of the sites showed a reduction in wrong rate error, but it was not statistically significant (*p* = 0.10) ▪ Compliance rates of using the smart pump were almost 100% in all sites ▪ In terms of compliance rates of using the smart pump and drug library, results were not significant, and smart pumps and drug library use error rates at the intervention sites slightly increased	NCC MERP severity rating: ▪ Smart pump/drug library not used - Pre-intervention: A (*n* = 4), B (*n* = 4), C (*n* = 109) - Post-intervention: A (*n* = 10), B (*n* = 2), C (*n* = 109) ▪ Wrong rate - Pre-intervention: A (*n* = 3), C (*n* = 45), D (*n* = 2) - Post-intervention: A (*n* = 1), C (*n* = 5) ▪ Pump setting error - Pre-intervention: C (*n* = 5) - Post-intervention: A (*n* = 1), C (*n* = 5)
Taylor (2019) [35]	▪ Medication errors with infusion pumps (*n* = 187, 91%) ▪ Factors contributing to rate error - Device maintenance (*n* = 4) - Malfunction (*n* = 4) - Patient behavior (*n* = 4) - Insufficient information (*n* = 16) - Pre-administration process problem (*n* = 18) - Tubing/connection (*n* = 19) - Programming (*n* = 122)	NA
Schilling (2022) [36]	▪ Among the 20 HAM selected in the first phase survey, 10 drugs had DRPs related to rate control of IV medications ▪ 9 DRPs were related to the rate control of IV medications ▪ 9 potential interventions for the DRP related to rate control of IV drugs	NA

*CI* Confidence interval, *DRP* Drug-related problems, *HAM* High-alert medications, *IV* Intravenous, *NA* Not applicable, *NCC MERP* National Coordinating Council for Medication Error Reporting and Prevention

Among the nine project or experiment studies, three showed a decrease in error rate as a result of the intervention [28, 31, 34]. Three studies developed interventions to reduce rate errors but did not report the frequency or incidence of rate errors [22, 24, 27]. A study reported the frequency of rate errors only after the intervention; the effect of the intervention could not be identified [30]. Also, three studies showed the severity of errors related to rate control of IV medications [24, 30, 34], two used NCC MERP severity ratings [30, 34], and one reported that all errors caused by smart IV pumps equipped with FMEA resulted in either temporary harm or no harm [24].

### Facilitators and barriers to rate control of IV medications

Table 3 presents the facilitators and barriers related to rate control of IV medications according to the NCC MERP taxonomy based on the 22 included studies. Sub-categories of human factors were classified as knowledge deficit, performance deficit, miscalculation of dosage or infusion rate, and stress. The sub-category of design factor was device. System-related contributing factors were classified as frequent interruptions and distractions, inadequate training, poor assignment or placement of HCPs or inexperienced personnel, policies and procedures, and communication systems between HCPs [14].

**Table 3:** Facilitators and barriers related to rate control of intravenous (IV) medication

Categories by NCC MERP^a^	Sub-categories	Facilitators	Barriers
Human factors	Knowledge deficit		- Lack of knowledge about vascular access related to patient posture [20] - Lack of knowledge about medication equipment [23] - Lack of drug knowledge about medications [24]
	Performance deficit		- Failure to check equipment properly [21] - Tubing misplacement [24, 35] - Monitoring inadequate [25] - Non-compliance with protocols and guidelines [2, 25] - Human handling errors with smart pumps [30]
	Miscalculation of dosage or infusion rate		- Error in infusion speed calculation [29]
	Stress (high-volume workload)		- High workload and distractions [23] - Error-prone ICU environment due to the heavy workload and complex critical care [37]
Design	Devices	- Expanding smart IV pump capabilities [26] - Monitoring pump programming at the system level [27] - Standardization of infusion pumps [22] - Using patient-controlled analgesia pumps and syringe drivers [28]	- Unexpected equipment faults [2, 20, 25, 35, 38] - Misassembly of an unfamiliar infusion pump [21] - Complex design of the equipment [23, 24] - Smart pumps that were not connected to electronic systems [30] - Incomplete drug libraries in smart pumps [33]
Contributing factors (system related)	Frequent interruptions and distractions		- A distracting environment in which nurses prepare medications [23] - Running multiple infusions at once [24, 27] - Air-in-line alarms or clearing air [24] - Error-prone ICU environment due to the heavy workload and complex critical care [37]
	Training	- Education on chemotherapy errors [22] - Mandatory end-user of smart IV pump training [24] - Education/training [36]	- Lack of appropriate training [23]
	Assignment or placement of a health care provider or inexperienced personnel	- Ward-based pharmacist [36]	- Nurses with < 6 years of experience [40]
	Policies and procedures	- Development of protocols for administering cytotoxic agents to nurses [22] - Providing information access [22] - Developing policy and procedure for standardizing overfill for infusion pump preparations and error follow-up [22] - Applying the FMEA method when introducing a smart IV pump [24] - Double-checks throughout the process [22, 24, 28, 36] - Using preprinted drug labels to identify tubing above and below the IV pump when running multiple infusions at once [24] - Continuous incidence reporting and subsequent prevention strategies [28] - Limiting the use of handwritten orders to emergency cases only [28] - Visual timers for IV pushes, no interruption zone with motion-activated indicators, speaking aloud, and reminder signage [31] - Use of point and calling method [39] - Use of infusion safety intervention bundle [34] - Standardized concentration and pre-printed label [36] - Standardized plan for dose tapering and infusion scheme [36]	- Absence of hospital policy that specifies a standard for KVO rate [30, 32] - Absence of a culture that promotes the use of smart pumps for all IV administrations [32, 33] - Medication orders that specified a duration rather than a rate [33] - Administering fluids for KVO at a low rate in anticipation of another infusion being needed [33] - Lack of automated infusion pumps [2]
	Communication systems between healthcare practitioners	- Communication with physicians in instances of doubt [28]	

*FMEA* Failure mode and effects analysis, *ICU* Intensive care unit, *IV* Intravenous, *KVO* Keep vein open, *NCC MERP* National Coordinating Council for Medication Error Reporting and Prevention

^a^Categories by NCC MERP: classified by medication error category according to NCC MERP [14]

#### Human factors

Among the barriers extracted from the 22 studies, 11 factors belonged to the “knowledge deficit,” “performance deficit,” “miscalculation of dosage or infusion rate,” and “stress (high-volume workload)” in this category. Half of these factors are related to the “performance deficit.” Barriers identified in two or more studies were tubing misplacement [24, 35] and non-compliance with protocols and guidelines [2, 25], all of which belonged to the “performance deficit.” Additionally, the high workload and environmental characteristics of the ICU, which corresponded to the “stress,” were also identified as barriers to rate control of IV medications [23, 37].

#### Design

Most factors in this category were related to IV medication infusion devices such as infusion pumps and smart pumps. In the study by Lyons et al., the use of devices, such as patient-controlled analgesia pumps and syringe drivers, was a facilitator of rate control of IV medications [33]. In addition to the use of these devices, the expansion of capabilities [26], monitoring programming [27], and standardization [22] were also facilitators. Unexpected equipment faults, a barrier, were identified in five studies [2, 20, 25, 35, 38]. Moreover, the complex design of the equipment [23, 24] and incomplete drug libraries in smart pumps [33, 35] were identified in two studies each. Factors such as the misassembly of an unfamiliar infusion pump [21] and smart pumps not connected to electronic systems [30] were also barriers.

#### Contributing factors (system related)

The factors belonging to the “frequent interruptions and distractions” in this category were all barriers. Specifically, running multiple infusions at once [24, 27], air-in-line alarms, or cleaning air [24] were identified as barriers. Among the facilitators of the “training,” there were education and training on the use of smart IV pumps [24] and chemotherapy errors [22]. There are two factors in the “assignment or placement of a HCP or inexperienced personnel,” where ward-based pharmacists were facilitators [36], but nurses with less than 6 years of experience were barriers [40]. The sub-category with the most factors was “policies and procedures,” where the facilitators extracted in the four studies were double-checks through the process [22, 24, 28, 36]. Among the barriers, two were related to keep-the-vein-open, which was identified in three studies [30, 32, 33]. The lack of automated infusion pumps [2], the absence of culture for use [32, 33], and problems in the drug prescription process [33] were also identified as barriers. Communication with physicians in instances of doubt identified was the only identified facilitator in the “communication systems between HCPs” [28].

### Resolutions for the barriers to rate control of IV medications

Table 4 presents the resolutions for the barriers to rate control of IV medications in the included studies. The suggested resolutions primarily belonged to the “contributing factors (system-related)” category. Resolutions in the “human factors” category were mainly related to the knowledge and performance of individual healthcare providers, and there were no studies proposing resolutions specifically addressing stress (high-volume workload), which is one of the barriers. Resolutions in the “design” category focused on the development [26, 30], appropriate use [24, 33], evaluation [26], improvement [24, 26, 30], and supply [23] of infusion pumps or smart pumps. Resolutions addressing aspects within the “contributing factors (system-related)” category can be classified into six main areas: interdisciplinary or inter-institution collaboration [23, 25, 28, 30, 34–37], training [24, 37, 40], implementation of policies or procedures [29, 31, 34, 35, 37, 39], system improvement [25, 30, 32], creating a patient safety culture [25, 37, 38], and staffing [2, 38].

**Table 4:** Resolutions for the barriers to rate control of intravenous (IV) medications suggested by the included studies

Categories by NCC MERP^a^	Resolutions for the barriers to rate control of IV medications
Human factors	- Appropriate monitoring and equipment check of the HCPs in the anesthetic department [20] - Supervision by a specialist and skilled assistance in the anesthetic department [20] - Rising anesthetists’ awareness of the continued integrity of vascular access systems [21] - Checking correct tip placement and labels of lines by the HCPs in the anesthetic department [21] - Establishing a stronger pharmacology knowledge base for nursing students and nurses [38] - Raising HCPs’ awareness to ensure appropriate setup, maintenance, and integration of smart pumps [35]
Design	- Supply products with a high safety standard by the manufacturers [23] - Short-term and long-term software and hardware changes to address failure modes with the new infusion pump [24] - The use of the appropriate site-specific drug profile through the new infusion pump [24] - Integration with barcoding and CPOE with the smart pump [26] - Incorporating real-time vital signs and laboratory data with the smart pump [26] - Automating monitoring and titration tasks with the smart pump [26] - Careful development and testing of smart pumps [26] - Drug dictionary in smart pumps reviewed by interdisciplinary committee members routinely and maintained up-to-date, evidence-based practice [30] - Assessing smart pump logs by the biomedical engineering department [30] - Investigating either physical or mechanical issues or human errors related to smart pumps by the biomedical engineering department [30] - Using smart pumps as part of an integrated system with barcode scanning and interfacing with electronic systems and reducing reliance on gravity feed [33]
Contributing factors (system related)	- Coordinated approach from practitioners, regulators, and the pharmaceutical industry [23] - Training for end users of the new infusion pump [24] - Healthcare FMEA between multiple institutions for discussion of best practices among pediatric oncology centers [25] - Different safety systems tailored for outpatient and inpatient chemotherapy settings [25] - Increased communication between adult and pediatric chemotherapy delivery systems to prevent similar errors from occurring [25] - A multidisciplinary approach that involves a change in hospital culture [28] - Collaboration with pharmacists to implement evidence-based interventions [28] - Increased training and supervision of new nurse graduates [40] - More obstetricians and nurses during the night shifts [2] - Improving nurses’ working procedures and implementing a clinical decision support tool that generates recommendations about adequate infusion rates [29] - Implementation of BCMA and e-MAR [29] - Integrated systems that are successfully implemented and utilized to get the full benefits of the safety system [30] - Reviewing reports related to smart pumps by the patient safety committee [30] - Hospital leadership working with a smart pump vendor to improve their products [30] - Changing work practices (taking more time for drug administration, using short infusions to administer some medication) [37] - Promoting a safety culture around medication, including drug preparation and administration [37] - Implementation of electronic prescribing systems, barcode medication administration, and pharmacist-led training program [37] - Multidisciplinary team with strong leadership endorsed by hospital managers for successful quality improvement [37] - Interventions that are more automated and less reliant on human memory and vigilance to prevent interruption-related errors [31] - Providing standard work conditions, such as a standard ratio of nurses to patients by hospital managers [38] - Improving the relationship between the nurses and physicians by hospital managers [38] - Facilitating the 24-h presence of clinical pharmacology experts for responding to medication questions by hospital managers [38] - Interoperability between currently implemented healthcare information technologies [32] - Implementation of point and calling methods and increasing compliance [39] - Development and implementation of the intervention bundle developed incorporating the expertise of the multidisciplinary research team [34] - A multidisciplinary approach when evaluating and procuring infusion pump [35] - A process to regularly collect safety-related-data, review the data, and create solutions to address pump-related concerns [35] - A multidisciplinary approach to identify and implement effective interventions to prevent medication-related harm in children [36]

*BCMA* Barcode medication administration, *CPOE* Computerized physician order entry, *e-MAR* Electronic medication administration record, *FMEA* Failure mode and effect analysis, *HCP* Healthcare providers

^a^Categories by NCC MERP: classified by medication error category according to NCC MERP [14]

## Discussion

This scoping review provides the most recent evidence on the attributes of errors, facilitators, and barriers related to rate control of IV medications. The major findings of this study were as follows: (1) there were a few intervention studies that were effective in decreasing the errors related to rate control of IV medications; (2) there was limited research focusing on the errors associated with IV medication infusion devices; (3) a few studies have systematically evaluated and analyzed the severity of errors associated with rate control of IV medications; and (4) the facilitators and barriers related to rate control of IV medications were identified by NCC MERP taxonomy as three categories (human factors, design, and system-related contributing factors).

Among the nine project or experiment studies, only two interventions showed statistically significant effectiveness for IV rate control [28, 31]. Six studies did not report the specific statistical significance of the intervention [22, 24, 27, 30, 37, 39], and one study found that the developed intervention had no statistically significant effect [34]. In another study, administration errors, including rate errors, increased in the experimental group and decreased in the control group [37]. IV rate control is a major process in medication administration that is comprehensively related to environmental and personal factors [3, 41]. According to previous studies, interdisciplinary or multidisciplinary cooperation is associated with the improvement in patient safety and decreased medical errors [42–44]. Seven of the included studies were also project or experiment studies that developed interventions based on an interdisciplinary or multidisciplinary approach [22, 24, 28, 30, 34, 37, 39]. Additionally, an effective intervention was developed by a multidisciplinary care quality improvement team [28]. Therefore, it is crucial to develop effective interventions based on an interdisciplinary or multidisciplinary approach to establish practice guidelines with a high level of evidence related to IV rate control.

Of the 22 included studies, three identified potential problems associated with the use of IV medication infusion devices [26, 32, 35], and four described the application of interventions or explored the effects of the intervention developed to reduce errors that occur when using IV medication infusion devices [24, 27, 34, 39]. IV medication infusion devices, such as infusion pumps and smart pumps, are widely used in healthcare environments and allow more rigorous control in the process of administering medications that are continuously infused [45]. Smart pumps are recognized as useful devices for providing safe and effective nursing care [15]. However, the use of IV medication infusion devices requires an approach different from traditional rate monitoring by counting the number of fluid drops falling into the drip chamber [9]. However, there exist many problems, such as bypassing the drug library, device maintenance, malfunction, tubing/connection, and programming in the use of IV medication infusion devices [32, 35]. None of the four studies that described the application of interventions or explored the effects of the intervention demonstrated statistically significant effects. All four studies had no control group [24, 27, 34, 39] and two studies had only post-test designs [24, 27]. Therefore, further research needs to be conducted to analyze errors in rate control related to IV medication infusion devices and develop effective interventions.

A few studies have systematically evaluated and analyzed the severity of errors associated with rate control of IV medications. Among the 12 studies that reported the severity of errors associated with rate control of IV medications, five studies used NCC MERP, an internationally validated and reliable tool for assessing error severity, and one study used the Severity Assessment Code (SAC) developed by the New South Wales Health Department. Six studies did not use tools to assess error severity. The term “error severity” means the degree of potential or actual harm to patients [46]. Evaluating the severity of medication errors is a vital point in improving patient safety throughout the medication administration process. This evaluation allows for distinguishing errors based on their severity to establish the development of risk mitigation strategies focused on addressing errors with the great potential to harm patients [47, 48]. Specifically, errors associated with rate control of IV medications were categorized as A to E on the NCC MERP and to groups 3 and 4 on the SAC. Additionally, errors associated with rate control of IV medications caused direct physical damage [2, 21] and necessitated additional medication to prevent side effects or toxicity [23]. Therefore, as errors in rate control of IV medications are likely to cause actual or potential harm to the patient, research systematically evaluating and analyzing error severity should be conducted to provide the basis for developing effective risk reduction strategies in the rate control of IV medications.

Facilitators and barriers were identified as human, design, and system-related contributing factors. Among the human factors, “performance deficit” included failure to check equipment properly, tubing misplacement, inadequate monitoring, non-compliance with protocols and guidelines, and human handling errors with smart pumps. Nurses play a major role in drug administration; thus, their monitoring and practices related to IV medication infusion devices can influence patient health outcomes [3, 49]. A major reason for the lack of monitoring was overwork, which was related to the complex working environment, work pressure, and high workload [3, 11, 49]. Moreover, two of the included studies identified high workload as a barrier to rate control of IV medications [23, 37]. Therefore, to foster adequate monitoring of rate control of IV medications, a systematic approach to alleviating the complex working environment and work pressure should be considered.

Most facilitators and barriers in the devices category were related to IV medication infusion devices. In particular, expanding pump capabilities [26], monitoring pump programming [27], standardization [22], and using a pump [33] can facilitate rate control of IV medications. However, unexpected equipment faults are significant barriers, as identified in five studies among the included studies [2, 20, 25, 35, 38]. Moreover, the design [23, 24], user-friendliness [21], connectivity to electronic systems [30], and completeness of drug libraries [33, 35] are factors that can affect rate control of IV medications. Therefore, it is important to improve, monitor, and manage IV medication infusion devices so that they do not become barriers. Moreover, because rate errors caused by other factors can be prevented by devices, active utilization and systematic management of devices at the system level are required.

Although there are many benefits of infusion and smart pumps for reducing errors in rate control of IV medications, they cannot be used in all hospitals because of the limitation of medical resources. The standard infusion set, which is a device for controlling the rate of IV medication by a controller [9], is widely used in outpatient as well as inpatient settings [32]. Devices for monitoring the IV infusion rate, such as FIVA™ (FIVAMed Inc, Halifax, Canada) and DripAssist (Shift Labs Inc, Seattle, USA), which can continuously monitor flow rate and volume with any gravity drip set, have been commercialized [33]. However, they have not been widely used in hospitals. Therefore, developing novel IV infusion rate monitoring devices that are simple to use, can be used remotely, and are affordable for developing and underdeveloped countries can help nurses to reduce their workloads in monitoring IV infusion rates and thus maintain patient safety.

Most facilitators and barriers were system-related contributing factors, most of which belonged to the “policies and procedures.” In four studies, the absence of hospital policies or culture related to rate control of IV medications was identified as a barrier [2, 30, 32, 33]. Medication errors related to incorrect rate control are problems that should be approached from macroscopic levels, such as via institutional policies and safety cultures. Therefore, large-scale research including more diverse departments and institutions needs to be conducted.

The second most common categories in system-related contributing factors were “frequent interruptions and distractions” and “training.” Although nurses experienced frequent interruptions and distributions during work, only one of the included studies was on interventions that were developed to create an environment with reduced interruptions [31]. Additionally, four studies found that education for nurses who are directly associated with medication administration is mandatory [22–24, 36]. Therefore, education and a work environment for safety culture should be created to improve IV rate control.

Based on resolutions for barriers to rate control of IV medications, key groups relevant to rate control of IV medications include HCPs, healthcare administrators, and engineers specializing in IV medication infusion devices. HCPs directly involved in the preparation and administration of IV medications need to enhance their knowledge of drugs, raise awareness for the importance of rate control of IV medications, and improve performance related to IV infusion device monitoring. Engineers specializing in IV medication infusion devices should develop these devices by integrating various information technologies used in clinical settings. Additionally, they should identify issues related to these devices and continuously enhance both software and hardware. Healthcare administrators play a crucial role in establishing and leading interdisciplinary or inter-institution collaborations. They should foster leadership, build a patient safety culture within the organization, and implement training, interventions, and policies for correct rate control of IV medications. Decreasing medication errors, including errors in IV rate control, is closely linked to the various key groups [50–53], and multidisciplinary collaboration is emphasized for quality care [54–57]. Therefore, each key group should perform its role and cooperate for appropriate IV rate control within a structured system.

This review has some limitations that should be considered. As there was no randomized controlled trial in this review, the causal relationship between wrong rate errors and their facilitators or barriers could not be determined. Moreover, only limited literature may have been included in this review because we included literature published in English and excluded gray literature. Since we did not evaluate the quality of the study, there may be a risk of bias in data collection and analysis. Despite these limitations, this study provides a meaningful assessment of published studies related to rate control of IV medications. This contribution will provide an important basis for new patient safety considerations in IV medication administration when determining future policies and device development.

## Conclusions

The findings of this review suggest that further research is needed to be conducted to develop effective interventions to improve the practice of IV rate control. Moreover, given the rapid growth of technology in medical settings, research on IV medication infusion devices should be conducted. Additionally, to establish effective risk reduction strategies, it is necessary to systematically evaluate and analyze the severity of errors related to the rate control of IV medications. Several facilitators and barriers to rate control of IV medications were identified in this review to ensure patient safety and quality care, interventions and policy changes related to education and the work environment are required. Additionally, the development of a device capable of monitoring the flow of IV medication is necessary. This review will be useful for HCPs, hospital administrators, and engineers specializing in IV medication infusion devices to minimize errors in rate control of IV medications and improve patient safety.

### Supplementary Information

**Additional file 1:** Preferred Reporting Items for Systematic Reviews and Meta-Analyses extension for Scoping Reviews (PRISMA-ScR) checklist.

**Additional file 2:** Search queries and strategies by electronic databases.

**Additional file 3:** Studies included in the data analysis.

## Data Availability

The corresponding author can provide the datasets that were utilized and/or examined during the present study upon reasonable request.

## Abbreviations

AE: Adverse event
HCP: Healthcare provider
ICU: Intensive care unit
IV: Intravenous
JBI: Joanna Briggs Institute
NCC MERP: The National Coordinating Council for Medication Error Reporting and Prevention
