# Supplementary material for: Attributes of errors, facilitators, and barriers related to rate control of IV medications: a scoping review
Source: Syst Rev. 2023 Dec 13;12:230. doi: 10.1186/s13643-023-02386-z (PMC10717502; doi:10.1186/s13643-023-02386-z)
Supplement: Supplementary file 2 — Additional file 2: Search queries and strategies by electronic databases. [file 13643_2023_2386_MOESM2_ESM.docx]

**Additional file 2. Search queries and strategies by electronic databases.**

1. PubMed

| Keyword | Search | Query | Result |
| --- | --- | --- | --- |
| IV therapy | #1 | (((("Drug Therapy"[Mesh]) OR "Fluid Therapy"[Mesh]) OR "Injections, Intravenous"[Mesh]) OR "Infusion Pumps"[Mesh]) OR "Administration, Intravenous"[Mesh] OR “Intravenous Therapy” OR “Intravenous Infusion” OR “Infusion Pumps” | 1,510,646 |
| Monitoring | #2 | ("Drug Monitoring"[Mesh]) OR "Medication Therapy Management"[Mesh] OR “Medication Monitoring” OR Drug Monitoring | 179,241 |
| Rate | #3 | time OR volume OR dose OR rate OR flow | 8,579,879 |
| Medication error | #4 | ((("Medical Errors"[Mesh]) OR "Patient Harm"[Mesh]) OR "Medication Errors"[Mesh]) OR "Drug-Related Side Effects and Adverse Reactions"[Mesh] OR mistakes | 260,754 |
| #1 AND #2 AND #3 AND # 4 | | | 971 |

2. Web of Science

| Keyword | Search | Query | Result |
| --- | --- | --- | --- |
| IV therapy | #1 | (TS=("drug therapy" OR "Fluid Therapy" OR "Injections, Intravenous" OR "Infusion Pumps" OR "Administration, Intravenous" OR Intravenous Therapy OR Intravenous Infusion OR Infusion Pumps)) | 166,426 |
| Monitoring | #2 | (TS=("Drug Monitoring" OR "Medication Therapy Management" OR “Medication Monitoring” OR Drug Monitoring)) | 112,343 |
| Rate | #3 | (TS= (time OR volume OR dose OR rate OR flow)) | 16,508,032 |
| Medication error | #4 | (TS=("Medical Errors" OR "Patient Harm" OR "Medication Errors" OR "Drug-Related Side Effects and Adverse Reactions" OR mistakes)) | 54,191 |
| #1AND #2 AND #3 AND #4 | | | 67 |

3. CINAHL

| Keyword | Search | Query | Result |
| --- | --- | --- | --- |
| IV therapy | #1 | (MH "Drug Therapy") OR (MH "Fluid Therapy") OR (MH "Injections, Intravenous") OR (MH "Infusion Pumps") OR (MH "Administration, Intravenous") OR Intravenous therapy OR Intravenous | 110,158 |
| Monitoring | #2 | (MH "Drug Monitoring") OR (MH "Medication Management") OR “Medication Monitoring” OR “Drug Monitoring” | 11,722 |
| Rate | #3 | time OR volume OR dose OR rate OR flow | 1,660,366 |
| Medication error | #4 | (MH "Medication Errors") OR "Patient Harm" OR (MH "Adverse Drug Event") OR “Medical Errors” OR mistakes OR "Drug-Related Side Effects and Adverse Reactions" OR mistakes | 76,487 |
| #1AND #2 AND #3 AND #4 | | | 44 |

4. EMBASE

| Keyword | Search | Query | Result |
| --- | --- | --- | --- |
| IV therapy | #1 | ‘Drug Therapy'/mj OR 'Fluid Therapy'/mj OR 'Intravenous Drug Administration'/mj OR 'Infusion Pump'/mj OR 'Administration, Intravenous'/mj OR intravenous OR 'Infusion Pumps' | 1,456,211 |
| Monitoring | #2 | ‘Drug Monitoring'/mj OR 'Medication Therapy Management'/mj OR 'Medication Monitoring' | 27,968 |
| Rate | #3 | time OR volume OR dose OR rate OR flow | 12,144,510 |
| Medication error | #4 | ‘Medication Error'/mj OR 'Patient Harm'/mj OR 'Adverse Drug Reaction'/mj OR mistakes OR ‘Medical Errors’ | 153,977 |
| #1AND #2 AND #3 AND #4 | | | 129 |
